# Open Surgical Conversion After Endovascular Aortic Aneurysm Repair: A Systematic Review and Meta-Analysis

**DOI:** 10.7759/cureus.57271

**Published:** 2024-03-30

**Authors:** Saeed S Alqahtani, Fahad K Aljaber, Bader Y Alsuwailem, Yazeed A AlMashouq, Bander G Alharbi, Riyadh H Masoud, Faisal A Albaqami

**Affiliations:** 1 Vascular Surgery, Charité – Universitätsmedizin Berlin, Berlin, DEU; 2 Vascular Surgery, Prince Sultan Military Medical City, Riyadh, SAU; 3 Vascular Surgery, King Fahad Medical City, Riyadh, SAU; 4 Vascular Surgery, Samsung Medical Center, Seoul, KOR

**Keywords:** elective conversion, emergency conversion, mortality, abdominal aortic aneurysm, late open conversion, endovascular aneurysm repair

## Abstract

Endovascular aneurysm repair (EVAR) is a preferred treatment for abdominal aortic aneurysms, though it comes with complications such as endoleaks and graft infections that may necessitate late open conversion (LOC). This systematic review and meta-analysis, drawing on studies from PubMed/MEDLINE, Embase, and the Cochrane Database of Systematic Reviews, aimed to evaluate the incidence, outcomes, and factors leading to LOC after EVAR. The analysis of 11 selected studies revealed a 5.3% incidence of LOC, with a patient cohort predominantly male (79%) and an average age of 73.5 years. The interval between initial EVAR and LOC was 35.1 months on average, with the Excluder device most frequently necessitating LOC. A notable 68% of endovascular salvage attempts before LOC failed. The study highlighted rupture and type I endoleak as the primary reasons for urgent LOC, which exhibited a 10-fold higher mortality rate compared to elective LOC. Elective LOC procedures had a 30-day mortality rate similar to primary elective open aneurysm repairs. These findings underscore the importance of vigilant post-EVAR patient monitoring and suggest that the methodological quality of underlying research should be considered in interpreting these results.

## Introduction and background

Endovascular aneurysm repair (EVAR) has revolutionized the treatment of abdominal aortic aneurysms (AAAs), offering a minimally invasive alternative to the traditional open surgical repair (OSR). This approach has been widely adopted due to its association with reduced perioperative morbidity, shorter hospitalization periods, and an improvement in early survival rates among patients [1]. The technique involves the placement of an endograft within the aorta to exclude the aneurysm from blood circulation, thereby preventing aneurysm growth and rupture [1].

Despite its advantages, EVAR is not without complications, including endoleaks, graft migration, infection, and aneurysm sac enlargement, which may compromise the integrity and success of the repair [2]. Such complications can necessitate open surgical conversion (OSC), a complex procedure involving the removal of the endograft and transition to an open repair method to secure aortic integrity [2]. The decision to proceed with OSC is multifaceted, influenced by the nature of the complication, patient-specific factors, and the potential for endovascular salvage [2].

The long-term management of EVAR patients is underscored by the necessity for vigilant surveillance to identify and address complications promptly. The indications for OSC, while clear in instances of endograft failure or significant complication, require careful consideration of the patient's overall health, the risks associated with open surgery, and the anticipated outcome improvements [3]. The emergence of fenestrated and branched endograft has expanded the applicability of EVAR to complex aneurysms, challenging the traditional boundaries of this technique and potentially altering the landscape of OSC indications [2,3].

The evolution of EVAR, marked by continuous improvement in endograft technology and procedural techniques, has significantly impacted the management strategies for aortic aneurysms [1]. However, the possibility of late complications necessitating OSC remains a pertinent concern, warranting a comprehensive review of the available evidence on this critical intervention [2,3]. The present study aims to systematically review and meta-analyze the indications, outcomes, and long-term impacts of OSC following EVAR, providing a consolidated foundation for clinical decision-making and identifying areas for future research. By elucidating the circumstances under which OSC is pursued, the outcomes associated with this intervention, and the factors predictive of its necessity, this review seeks to inform clinical practice, enhance patient counseling, and ultimately contribute to the refinement of aortic aneurysm management protocols.

## Review

Methods

Literature Review

This systematic review was conducted following the guidelines outlined in the PRISMA (Preferred Reporting Items for Systematic Reviews and Meta-Analyses) 2020 statement and the AMSTAR (A Measurement Tool to Assess Systematic Reviews) guidelines. Our objective was to comprehensively aggregate and analyze data on OSC after EVAR, focusing on late conversions and their associated outcomes. We systematically searched PubMed/MEDLINE, Embase, and the Cochrane Database of Systematic Reviews using a combination of keywords related to EVAR and aortic aneurysms, including endovascular-related terms (endovasc*, endoprosth*, stent-graft, "stent graft*", endograft, EVAR, EVAS, seal, Nellix) and aneurysm-related terms (aneurysm*, aort*). Our search was limited to articles published in English without imposing any time restrictions. Duplicate entries identified across the databases were removed with the aid of RefWorks software.

Inclusion and Exclusion Criteria

For a study to be eligible for inclusion, it needed to meet specific criteria centered around the theme of endograft explantation after EVAR. The studies had to report on instances where the explantation occurred more than 30 days following the initial implantation, a scenario classified as a late open conversion (LOC). Additionally, these studies were required to provide data on the 30-day mortality rate, with a clear differentiation between urgent and elective conversions. The criteria specified that urgent conversions encompassed cases such as aortoduodenal fistulas, stent graft infections, and interventions for symptomatic or ruptured aneurysms. We prioritized the most recent publication in instances of data overlap to ensure the most current data was analyzed.

**Figure 1:**
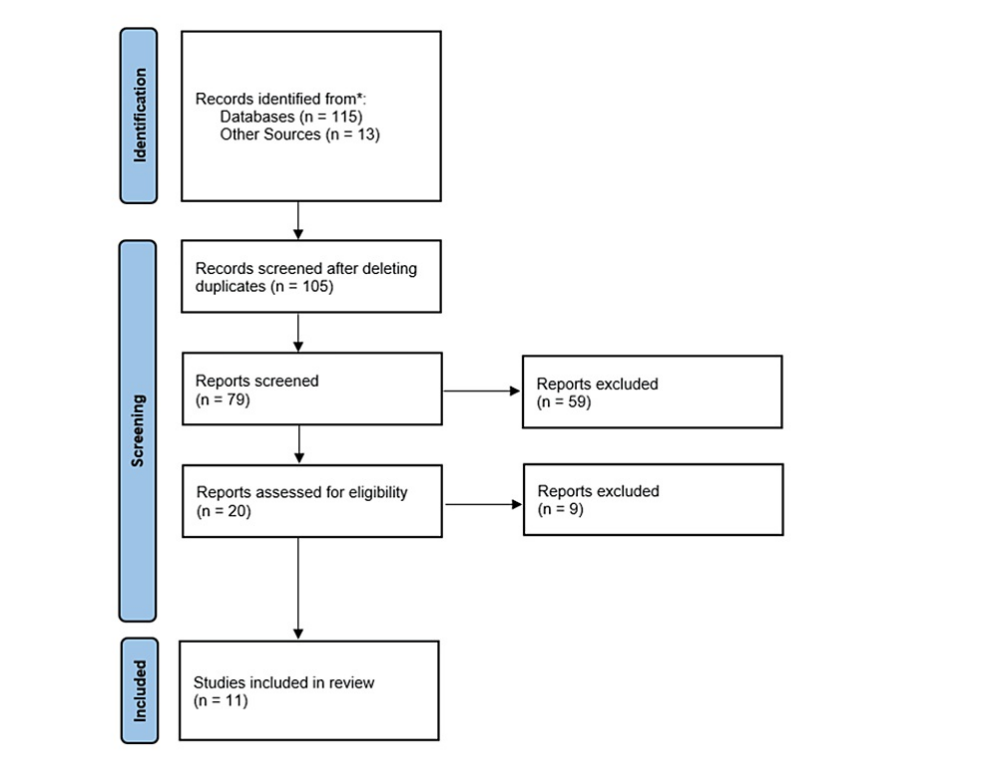
PRISMA 2020 flow diagram PRISMA: Preferred Reporting Items for Systematic Reviews and Meta-Analyses

On the other hand, the review excluded certain types of publications to maintain a focus on relevant, high-quality evidence. These excluded types were case reports or series involving fewer than 10 patients, purely technical descriptions of the conversion procedure without substantial outcome data, review articles, and letters to the editor. This exclusion criteria aimed to ensure that the collected data was both significant in terms of patient numbers and focused on the outcomes of interest rather than procedural descriptions or preliminary observations (Figure 1).

Data Collection Process

The process for selecting relevant studies involved two reviewers independently screening the titles and abstracts for potential inclusion based on the aforementioned criteria. This step required a consensus between the reviewers for a study to advance to the full review stage. In cases of disagreement, a discussion or the input of a third reviewer was sought to reach a decision. The selected studies then underwent a full-text review to confirm eligibility, during which detailed data on several aspects of the LOC process were extracted. These aspects included but were not limited to the indications for endograft explantation, the type of endograft used, the surgical approach for explantation, the timing of the conversion relative to the initial implantation, and the diameter of the AAA at the time of conversion. The studies also provided data on the immediate outcomes, specifically the 30-day mortality rates, and categorized these outcomes based on whether the conversion was performed on an urgent basis or was elective. Additionally, information regarding the publication year, study design, duration, and patient demographics including prevalent comorbid conditions was gathered to facilitate a comprehensive analysis of the findings.

Outcome Measures and Data Analysis

Our analysis focused on the outcomes of OSR following EVAR, specifically targeting infectious native aortic aneurysms (INAA). We employed the Newcastle-Ottawa Scale and the Moga score to assess the methodological quality of the included studies. Data were synthesized using random-effects models to calculate pooled metrics, reflecting both urgent and elective conversion outcomes.

Assessment of Risk of Bias in Included Studies

Evaluating the risk of bias in each included study is crucial, irrespective of the study type (e.g., reviews, randomized clinical trials, observational studies, etc.). Rather than creating a new assessment tool, we utilized published, structured, and ideally validated risk-of-bias assessment tools. This approach ensures that evaluations are thorough, consistent, and as objective as possible. The selection of an inappropriate tool or the failure to use a structured tool could introduce bias. The necessary steps have been undertaken to mitigate this risk (Table 1).

**Table 1:** Summary of risk of bias assessment Domains: D1: bias due to confounding; D2: bias in selection of participants; D3: bias in classification of interventions; D4: bias due to deviations from intended interventions; D5: bias due to missing data; D6: bias in measurement of outcomes; Overall: overall risk of bias Judgment: + low, - some concerns, X high concern Included studies: Ben Abdallah et al. [4], Arya et al. [5], Brinster et al. [6], Chaar et al. [7], Ferrero et al. [8], Joo et al. [9], Klonaris et al. [10], Kansal et al. [11], Bonardelli et al. [12], Perini et al. [13], Turney et al. [14]

Study	D1	D2	D3	D4	D5	D6	Overall
Ben Abdallah et al., 2017 [4]	−	+	+	−	−	−	−
Arya et al., 2013 [5]	−	−	+	+	+	+	+
Brinster et al., 2011 [6]	−	+	−	+	−	+	+
Chaar et al., 2012 [7]	−	+	+	−	+	−	−
Ferrero et al., 2013 [8]	X	−	+	+	−	−	−
Joo et al., 2019 [9]	−	+	−	+	+	+	+
Klonaris et al., 2014 [10]	−	+	+	−	−	+	+
Kansal et al., 2018 [11]	−	+	−	−	+	+	+
Bonardelli et al., 2018 [12]	X	+	+	+	−	+	+
Perini et al., 2017 [13]	−	−	+	−	+	−	+
Turney et al., 2014 [14]	−	+	+	−	+	−	+

Results

Overview of Studies and Patient Demographics

Our comprehensive analysis integrated data from 11 studies [4-14], revealing that the cumulative incidence of LOC post-EVAR was identified to be 5.3% (95% CI: 3.1-7.4%). It is noteworthy that specific incidence rates by center were not disclosed by Millon et al. [15], who reported aggregate EVAR operations across eight centers, and Kong et al. [16] presented participant totals without detailing the conversion rate. The clarity regarding the volume of EVAR procedures conducted during the study periods was lacking or ambiguously reported in several instances. The demographic analysis indicated an average patient age of 73.5 years (95% CI: 72.3-74.7), with males constituting 79% of the study population. The prevalence of hypertension was observed in 50.7% of patients (95% CI: 47.2-54.2%), coronary artery disease in 39.6% (95% CI: 36.2-43%), smoking in 25.5% (95% CI: 22.5-28.6%), and diabetes mellitus in 12.3% (95% CI: 10-14.5%), despite a considerable number of patients not reporting on these conditions (Table 2).

**Table 2:** Summary of studies AAA: abdominal aortic aneurysm; BS: bilateral subcostal; EVARs: endovascular aneurysm repairs; IN: indecipherable or murky; MC: multi-center; MINORS: Methodological Index for Non-Randomized Studies; ND: no data available; NR: not reported; TL: thoracophrenic laparotomy; TP: transperitoneal; R: retrospective; RP: retroperitoneal; SC: single center

Author	Study type	Total late EVARs performed (conversion %)	Number of late conversion	Number of elective cases
Ben Abdallah et al., 2017 [4]	R + SC	338 (9.2)	31	19
Arya et al., 2013 [5]	R + SC	270 (14.4)	39	30
Brinster et al., 2011 [6]	R + SC	1273 (1.6)	21	16
Chaar et al., 2012 [7]	R + two centers	Total: 1682 (2.6); center 1: 1549 (2.6); center 2: 133 (3.0)	44	19
Ferrero et al., 2013 [8]	R + SC	415 (4.8)	20	13
Joo et al., 2019 [9]	R + SC	566 (5.3)	30	24
Klonaris et al., 2014 [10]	R + SC	442 (4.1)	18	15
Kansal et al., 2018 [11]	R + SC	1060 (1.5)	16	10
Bonardelli et al., 2018 [12]	R + SC	435 (5.5)	24	21
Perini et al., 2017 [13]	R + SC	NR	28	21
Turney et al., 2014 [14]	R + SC	1881 (5.3)	100	71

Timeframe and Study Duration

The studies spanned from 2005 to 2016, covering a substantial period of up to 21 years. The time interval from initial EVAR implantation to conversion averaged 35.1 months (95% CI: 30.4-39.8 months), ranging broadly from one to 228 months, albeit data for 41 patients were not accounted for.

Indications for Conversion

The predominant reason for LOC was endoleak, accounting for 62.3% (95% CI: 58.7-65.4%) of cases, followed by aneurysm rupture (11.1%; 95% CI: 8.8-13.2%), infection (8.2%; 95% CI: 6.3-10.1%), and endotension (5.8%; 95% CI: 4.2-7.4%). Specifically, 80.7% of elective conversions were attributed to endoleaks, while ruptures were responsible for 48.1% of urgent conversions. The median aneurysm size at conversion was reported as 61.5 mm, ranging from 58.3 mm to 70.8 mm, with data missing for 39.2% of the patients. The Excluder endograft emerged as the most frequently explanted device (16.2%; 95% CI: 13.6-18.8%), followed closely by Talent and Zenith endograft.

Surgical Techniques and Approaches

Transperitoneal surgery was the predominant approach, employed in 74% of cases (95% CI: 70.9-77%), with retroperitoneal access utilized in 15% (95% CI: 12.6-17.5%). Other surgical techniques, such as subcostal and transverse incisions, were less commonly used. The surgical approach details were not recorded for 9.5% of the procedures.

Postoperative Complications and Interventions

The postoperative complications varied by the type of endograft used. Notably, the AFX endograft was linked to limb occlusion, while the Cook Zenith and Ovation endograft reported no postoperative complications. Endoleaks of types I and II were associated with Endologix and Endurant II endografts, respectively, and the Excluder endograft was implicated in renal failure and bowel ischemia incidents. Before opting for OSC, various endovascular interventions, such as embolization and stent-graft extensions, were attempted to address complications like type II endoleaks or stenosis.

**Figure 2:**
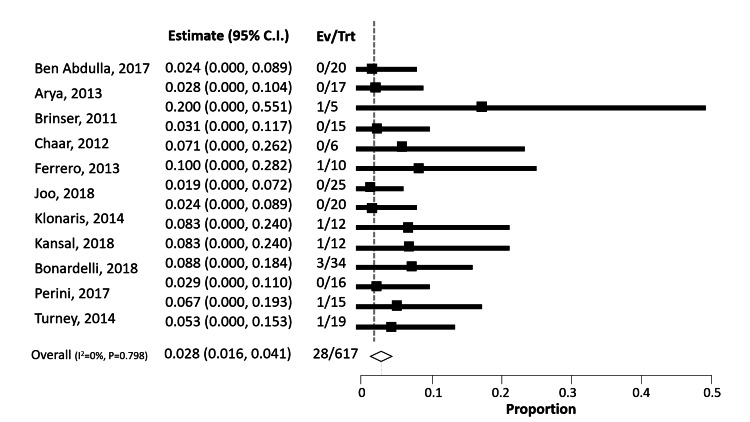
Forest plot illustrating the outcomes in the studies included in the meta-analysis The studies included in this analysis are as follows: Ben Abdallah et al. [4], Arya et al. [5], Brinster et al. [6], Chaar et al. [7], Ferrero et al. [8], Joo et al. [9], Klonaris et al. [10], Kansal et al. [11], Bonardelli et al. [12], Perini et al. [13], Turney et al. [14]

This meta-analysis found that the 30-day mortality rate was 10 times higher for late urgent conversions in failed EVAR (28.3% vs. 2.8%) compared to late elective conversions. Endoleaks continue to be the most problematic aspect of EVAR, as they can occur at any time. Type I (n=207; 26.2%) and II (n=99; 12.5%) endoleaks were reported as the most common types in the included investigations (Figure 2).

## Conclusions

This study underscores the pivotal distinctions between emergency and elective LOCs following EVAR, revealing that the precipitants of emergency interventions, primarily aneurysm rupture and type I endoleak, significantly elevated mortality rates. Specifically, the mortality rate associated with emergency late conversions is markedly higher, by a 10-fold increase, compared to elective late conversions. Interestingly, the mortality rate of elective late conversions parallels that observed in primary elective open AAA repairs at the 30-day postoperative mark. Nonetheless, the interpretation of these outcomes necessitates a cautious approach, taking into account the quality and robustness of the existing evidence base. Additionally, it is noteworthy that post-EVAR, certain patients may require further interventions, such as coil embolization or balloon angioplasty, to manage concurrent conditions, highlighting the complexity and individualized nature of care required in these scenarios.
